# Processed Dietary Fiber Partially Hydrolyzed Guar Gum Increases Susceptibility to Colitis and Colon Tumorigenesis in Mice

**DOI:** 10.21203/rs.3.rs-5522559/v1

**Published:** 2024-12-09

**Authors:** Sangshan Tian, Umesh K Goand, Devendra Paudel, Giang V. Le, Amit K. Tiwari, K. Sandeep Prabhu, Vishal Singh

**Affiliations:** The Pennsylvania State University; The Pennsylvania State University; The Pennsylvania State University; The Pennsylvania State University; University of Arkansas for Medical Sciences; The Pennsylvania State University; The Pennsylvania State University

**Keywords:** Inflammatory bowel disease, Fermentable dietary fiber, Intestinal inflammation, Colorectal cancer, Oncogenes

## Abstract

The vital role of naturally occurring dietary fibers (DFs) in maintaining intestinal health has fueled the incorporation of isolated DFs into processed foods. A select group of soluble DFs, such as partially hydrolyzed guar gum (Phgg), are being promoted as dietary supplements to meet recommended DF intake. However, the potential effects of regular consumption of these processed DFs on gastrointestinal health remain largely unknown. The present study assessed the impact of Phgg on the development of intestinal inflammation and colitis-associated colon carcinogenesis (CAC). Wild-type C57BL/6 mice were fed isocaloric diets containing either 7.5% Phgg and 2.5% cellulose (Phgg group) or 10% cellulose (control) for four weeks. To induce colitis, a subgroup of mice from each group was switched to 1.4% dextran sulfate sodium (DSS) in drinking water for seven days. CAC was induced in another subgroup through a single dose of azoxymethane (AOM, 7.5 mg/kg i.p.) followed by three DSS/water cycles. To our surprise, Phgg feeding exacerbated DSS-induced colitis, as evidenced by body weight loss, disrupted colonic crypt architecture, and increased pro-inflammatory markers accompanied by a decrease in anti-inflammatory markers. Additionally, Phgg feeding led to increased colonic expression of genes promoting cell proliferation. Accordingly, extensive colon tumorigenesis was observed in Phgg-fed mice in the AOM/DSS model, whereas the control group exhibited no visible tumors. To investigate whether reducing Phgg has a distinct effect on colitis and CAC development, mice were fed a low-Phgg diet (2.5% Phgg). The low-Phgg group also exhibited increased colitis and tumorigenesis compared to the control, although the severity was markedly lower than in the regular Phgg (7.5%) group, suggesting a dose-dependent effect of Phgg in colitis and CAC development. Our study reveals that Phgg supplementation exacerbates colitis and promotes colon tumorigenesis, warranting further investigation into the potential gastrointestinal health risks associated with processed Phgg consumption.

## 1. Introduction

Colorectal cancer (CRC) is the third-leading cause of cancer-related deaths in the United States, posing a significant public health challenge^1^. According to the data from Global Cancer Observatory (GCO), approximately 1.9 million new cases of CRC were diagnosed worldwide in 2022^2^. Over 95% of CRCs are adenocarcinomas. The transformation of an intestinal polyp into cancer occurs through the adeno-to-carcinoma sequence, a series of genetic changes involving proto-oncogene mutations and altered expression of tumor suppressor and cell survival genes that promote tumor growth^3^. Factors contributing to CRC include aging, genetic predisposition, environmental influences, and prolonged intestinal inflammation^4^. Colitis-associated colorectal cancer (CAC) is defined as a form of CRC that is commonly seen in patients with inflammatory bowel disease (IBD). Prolonged colonic inflammation significantly increases the risk of CAC compared to healthy individuals^5, 6^. Although the detailed molecular pathways remain underexplored, the IBD patients displaying chronic, hyperactive immune responses are at an elevated risk of developing CAC^5^. Higher consumption of fiber-rich whole foods, such as fruits and vegetables improve overall well-being, including gastrointestinal health^7^. Dietary Guidelines for Americans recommend increasing fiber intake primarily from whole and minimally processed foods. Since only about 10% of Americans meet their dietary recommendation of fruits and vegetables^8^, incorporating isolated dietary fibers (DFs) into processed foods presents a potential strategy to meet their daily intake. This approach, in fact, is marketed as enhanced nutritional value of these foods and to help meet fiber intake.

DFs are edible complex carbohydrates that are resistant to human digestion. DFs broadly are classified into insoluble and soluble based on their solubility in water. Insoluble fibers like cellulose are generally resistant to fermentation by gut bacteria in both humans and mice. Despite limited bacterial breakdown, they offer various health benefits, including increased stool bulk and a laxative effect^9^. Soluble DFs such as inulin and partially hydrolyzed guar gum (Phgg) are readily fermented by both human and murine gut microbiota. These soluble DF also offer numerous benefits to the host’s metabolic and gastrointestinal (GI) health^10^. Improvements in intestinal health markers, such as enhanced gut barrier function^11^, reduced levels of pro-inflammatory cytokines^12^, and increased colonic cell proliferation^13, 14, 15^, upon consuming these soluble DFs, primarily stemmed from interventional studies conducted in cohorts of healthy rodents and human populations. Whether DF supplementation exerts similar effects during ongoing intestinal inflammation remains largely unknown and has begun to be unraveled recently. These recent studies have produced conflicting results regarding the impact of refined DFs on intestinal health. A subset of these studies suggests beneficial effects on intestinal inflammation^16, 17, 18, 19^ while another subgroup raises concerns about potential adverse effects on both intestinal inflammation and colon tumorigenesis^20, 21, 22, 23, 24^. A comprehensive evaluation of refined DFs that are being incorporated into the ultra-processed food and promoted as supplements, is necessary to determine their impact on intestinal health, especially during periods of ongoing inflammation. In this study we evaluated Phgg, a soluble DF widely used in the food industry as a food thickener and emulsifier^25^, and available as a supplement, on colonic inflammation and the markers of cell survival and proliferation. Additionally, we evaluated the effect of Phgg on colon tumorigenesis using a colitis-associated colon cancer model^26^.

## 2. Results

### 2.1. Diet containing refined Phgg exacerbated colonic inflammation

**Figure 1:**
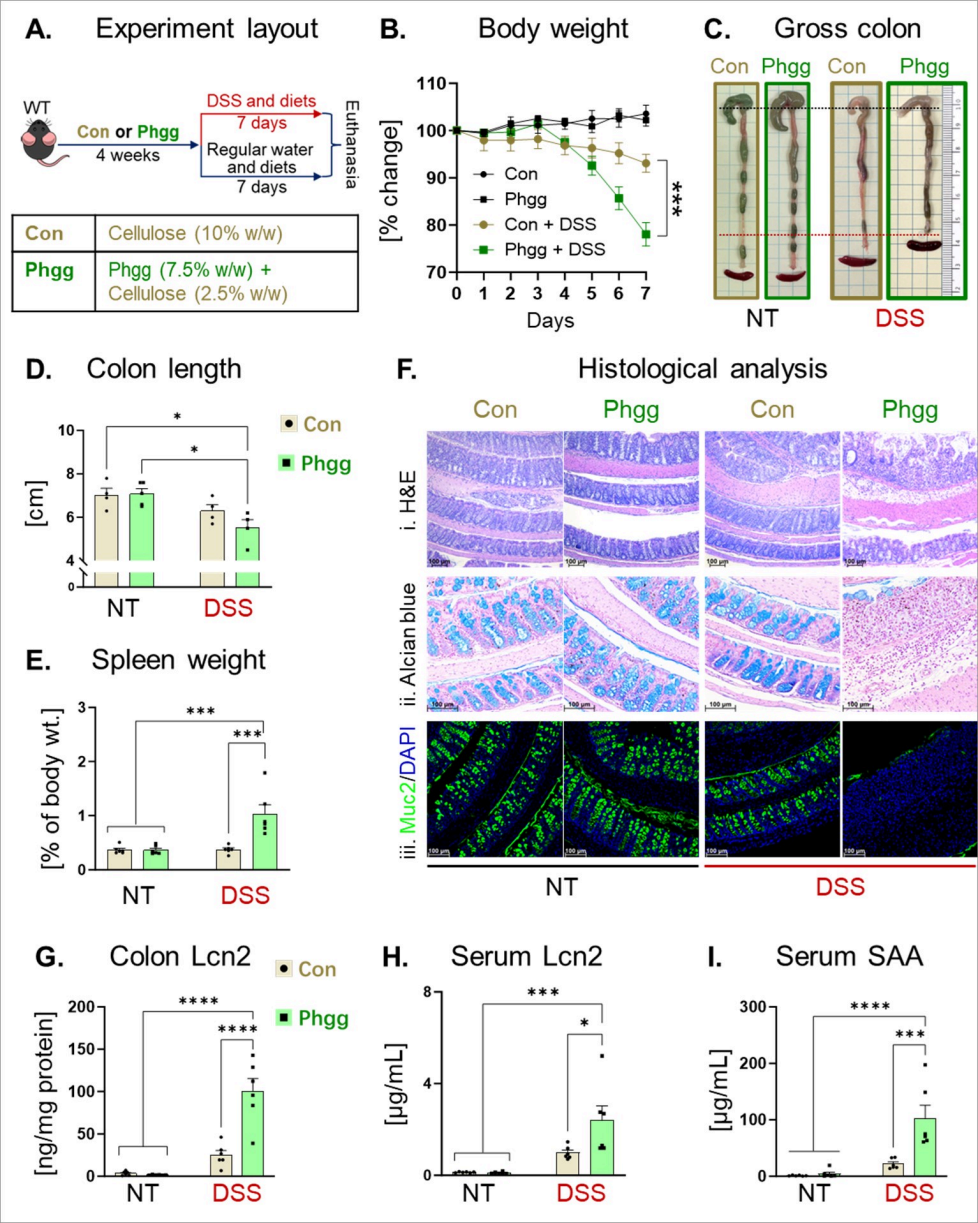
Phgg exacerbates DSS-induced colitis. **A.** Experimental layout. **B.** Percent change in body weight (b. wt.) during the DSS intervention period. **C.** Representative gross colon images. **D.** Colon length. **E.** Spleen weight (as a percent of the b. wt. on euthanasia day). **F.** Representative images of (i) H&E-stained, (ii) alcian blue-stained colon sections (original magnification, x100), and (iii) immunohistochemical staining for mucin 2 (Muc2; green), with DAPI (blue) to visualize the nucleus (original magnification, x200). **G.** Colon Lcn2. Serum levels of **H.** Lcn2 and **I.** SAA. Values are presented as mean ±SEM (B, D-E, and G-I). *p < 0.05, **p < 0.01, ***p < 0.001, ****p < 0.0001.

Since a subset of patients with IBD reports heightened inflammation after consuming certain DF^20^, we examined the effect of processed DF Phgg on colitis in a mouse model of acute colitis. WT mice, one-week post-weaning, were maintained on a Phgg-containing diet (7.5% w/w Phgg, 2.5% w/w cellulose) or a control diet (10% w/w cellulose) for four weeks. Subsequently, mice were divided into two groups receiving either regular water (no treatment, NT) or were administered dextran sulfate sodium-containing water (DSS; 1.4% w/v) for seven days (Fig. 1A). Although no significant differences were found in the body weight among the NT groups, Phgg-fed mice challenged with DSS lost approximately 15% more weight than the control-DSS group (Fig. 1B). Furthermore, shortened colon length and increased spleen weights were observed in Phgg-DSS group compared to rest of the groups (Fig. 1C-E). Histological analysis demonstrated epithelial damage, loss of crypt structure, and immune cell infiltration within the submucosal layer in DSS-challenged mice maintained on Phgg (Fig. 1Fi). The Phgg-fed group also exhibited a substantial loss of goblet cells and reduced mucin 2 secretion compared to the control group in the DSS-intervention group. (Fig. 1Fii-iii). In line, mice in Phgg-DSS group showed significant elevation in both colonic and systemic lipocalin-2 (Lcn2), a biomarker of colonic inflammation, and serum amyloid A (SAA) (Fig. 1G-I). Remarkably, Phgg feeding in the experimental group without colitis displayed comparable levels of intestinal health markers, including mucin expression and immune markers. This suggests that Phgg supplementation alone does not adversely impact intestinal health but fuels ongoing inflammation and worsens colitis.

### 2.2. Phgg supplementation alters colonic immune markers favoring inflammation

**Figure 2:**
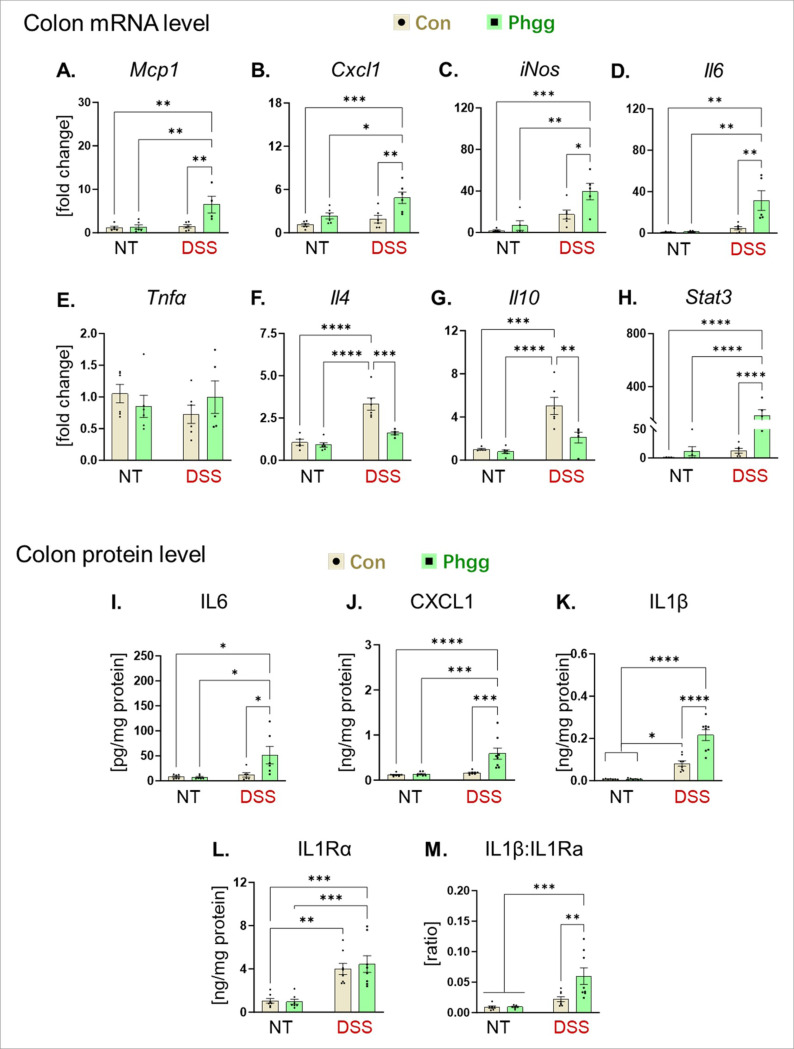
Phgg-fed group displays increased pro- and reduced anti-inflammatory markers upon DSS intervention The colonic tissue was obtained from no treatment (NT) and DSS intervention (DSS) groups and analyzed for mRNA expression (via qPCR) and protein level (via ELISA). **A.** *Monocyte chemoattractant protein 1*(*Mcp1*), **B.** *CXC motif chemokine ligand 1* (*Cxcl1*), **C.** *Inducible nitric oxide synthase* (*iNos*), **D.** *Interleukin 6* (*Il6*), **E.** *Tumor necrosis factor alpha* (*Tnfα*), **F.** *Il4,* and **G.** *Il 10*. **H.** *Signal transducer and activator of transcription 3* (*Stat3*). (**I-M**) Protein level was estimated via ELISA and normalized by total colon protein concentration. **I.** IL6, **J.** CXCL1, **K.** IL1β, **L.** IL1 receptor antagonist (IL1Ra), and **M.** IL1β to IL1Ra ratio. Values are presented as mean ±SEM. *p < 0.05, **p < 0.01, ***p < 0.001, ****p < 0.0001.

To determine the factors that worsened colonic inflammation in the Phgg-fed group, we measured the expression of chemokines and cytokines in the colon. Increased colonic mRNA expression of monocyte chemoattractant protein-1 (*Mcp1*) and C-X-C motif chemokine ligand-1 (*Cxcl1*) in Phgg-fed mice treated with DSS (Fig. 2A-B) suggested increased infiltration of immune cells. This was evidenced in H&E-stained sections, which revealed extensive inflammatory cell infiltration in the colonic mucosa and submucosa compared to DSS-treated control. In line, we observed elevated mRNA transcripts of the pro-inflammatory markers inducible nitric oxide synthase (*iNos*) and interleukin-6 (*Il6*) specifically in the Phgg + DSS group compared to the remaining groups (Fig. 2C-D). The expression of tumor necrosis factor-alpha (*Tnfα*) remained unaltered across the groups (Fig. 2E). Most notably, the mRNA levels of anti-inflammatory cytokines *Il-4* and *Il-10* were augmented in the DSS-treated control group but not in the Phgg + DSS group (Fig. 2F-G). In fact, Phgg + DSS group displayed reduced colonic expressions of *Il-4* and *Il-10*. The colonic mRNA expression data suggest that Phgg supplementation not only promoted the expression of pro-inflammatory molecules but also reduced the levels of anti-inflammatory molecules.

IL-6 is considered both an intestinal immune activity modulator and a tumorigenesis promoter^27, 28^. The proliferative and survival effects of IL-6 are largely mediated by signal transducer and activator of transcription 3 (STAT3)^29^. Intriguingly, the colonic mRNA level of *Stat3* was exclusively augmented in the Phgg + DSS group (Fig. 2H). We further examined colonic protein levels of the pleiotropic cytokine IL-6, the chemokine CXCL1, the pro-inflammatory cytokine IL-1β, and its physiological antagonist, the IL-1 receptor antagonist (IL-1Ra), using ELISA. We found increased colonic levels of IL-6, CXCL1, and IL-1β in the Phgg + DSS group compared to the DSS-treated control (Fig. 2I-K). IL-1Ra inhibits inflammation mediated by IL-1β by blocking its binding to its receptor, IL-1R1. Therefore, to assess the IL-1β activity, we examined the colonic IL-1β/IL-1Ra ratio, which was markedly elevated in the DSS-treated Phgg-fed group (Fig. 2L-M), suggesting heightened IL-1β-mediated immune activity in this group. Altogether, these data indicate an imbalanced intestinal inflammatory milieu arising from elevated pro-inflammatory factors and reduced anti-inflammatory cytokines, which contributed to exacerbating colitis in the Phgg-fed group.

### 2.3 Phgg induces aberrant expression of intestinal barrier function and cell proliferation markers

**Figure 3:**
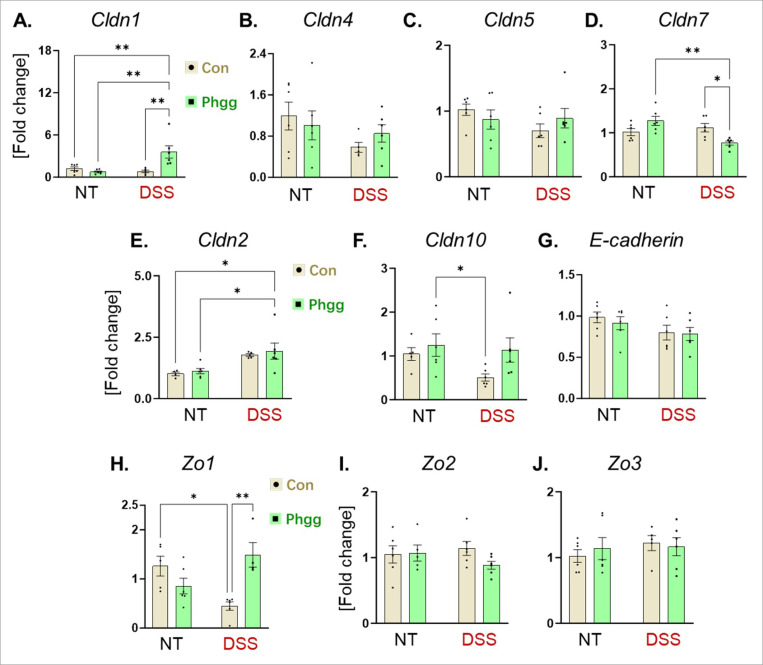
Phgg-fed mice exhibit differential gene expression of tight junction proteins in colon The expression of tight junction proteins was estimated at mRNA level via (qPCR) in colon tissues. **A.** *Claudin 1* (*Cldn1*), **B.** *Cldn4*, **C.** *Cldn5*, **D.** *Cldn7*, **E.** *Cldn2*, and **F.** *Cldn10*. **G.** *E-cadherin.* ***H.*** *Zonula occludes 1* (*Zo1*), **I**. *Zo2*, and **J.** *Zo3*. Values are presented as mean ±SEM. *p < 0.05, and **p < 0.01.

Tight junction (TJ) proteins maintain the intestinal barrier integrity protecting against gut microbial invasion^30^ and regulate the mucosal repair^31, 32^. Thus, colonic mRNA transcripts of TJ proteins were assessed. Among the barrier-forming claudins (Cldn1, 4, 5, and 7), the mRNA level of *Cldn1* was significantly elevated in the Phgg + DSS group (Fig. 3A). This finding aligns with human IBD specimens, which exhibited increased CLDN1 in ulcerative colitis (UC) colon compared to non-disease colon^33^. Concurrently, we observed reduced colonic expression of *Cldn7* (Fig. 3D), whose deficiency is shown to increase susceptibility to colitis and associated colon tumorigenesis^34, 35, 36^. The colonic levels of *Cldn4*, and *Cldn5* remain unaltered across the groups (Fig. 3B-C). Next, we examined the colonic expression of pore-forming claudins, *Cldn2* and *Cldn10*. Phgg-fed mice displayed increased level of *Cldn2*^37^ in colitis group (Fig. 3E). Data from mucosal biopsy specimens from human patients with UC show a similar pattern, with low CLDN2 expression in normal colon and an increase in CLDN2 in mucosal specimens from human patients with UC^38^. Another pore-forming claudin, *Cldn10*^39^ exhibited an increasing trend in the Phgg + DSS group, although the data did not reach statistical significance (Fig. 3F). The expression of E-cadherin^40^, which regulates the incorporation of claudins into tight junctions, was comparable across all four groups (Fig. 3G). We next evaluated the zonula occludens (ZO −1, −2, and − 3)—ZOs are membrane-associated cytosolic scaffolding proteins that facilitate assembly of TJ proteins, including claudins^41^. Among the three types of ZOs, only *Zo1* expression was significantly altered (increased) in Phgg-fed mice challenged with DSS, indicating activation of the mucosal repair mechanism^42^ in response to epithelial injury (Fig. 3H-J).

**Figure 4:**
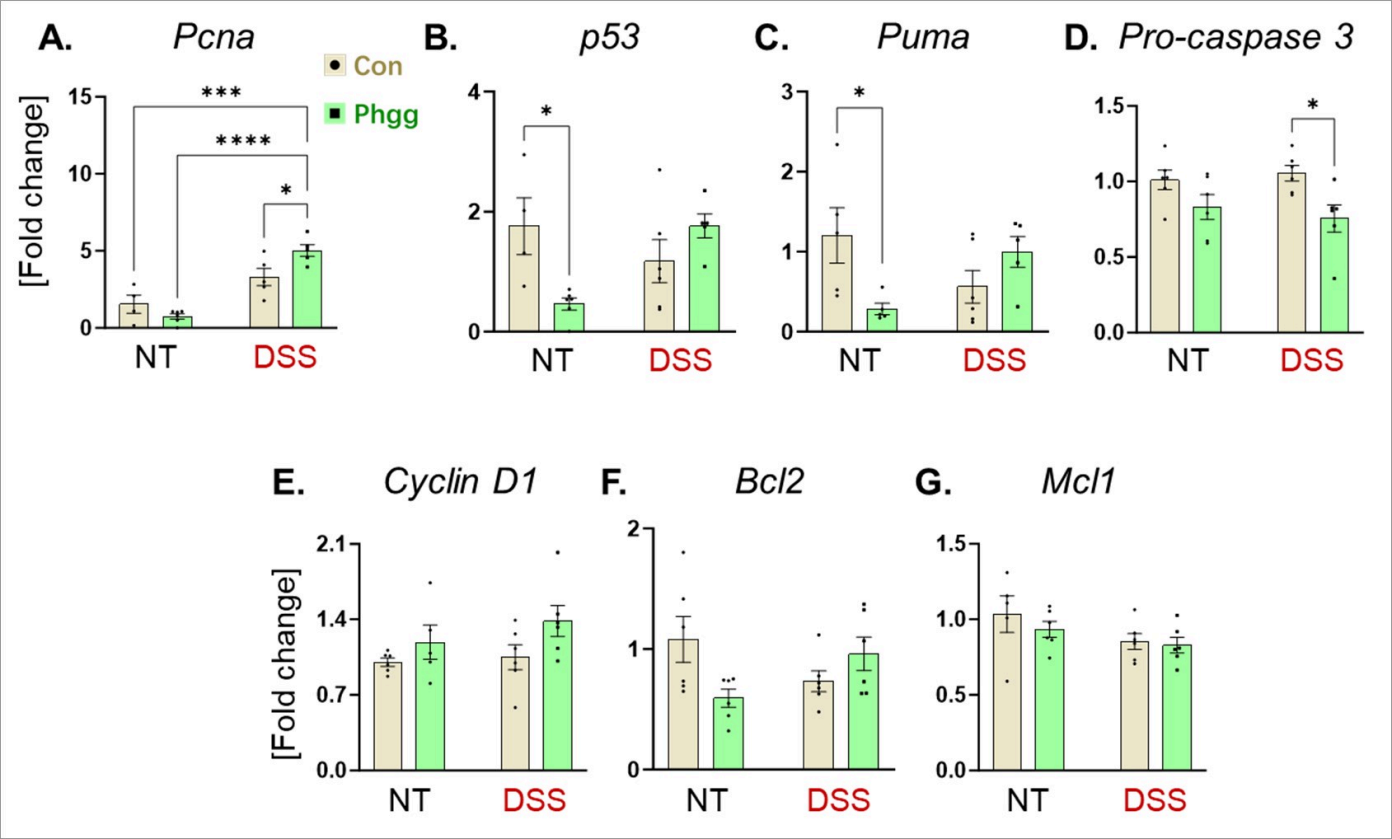
Phgg feeding alters colonic expression of genes related to proliferation and apoptosis Colonic mRNA levels of **A.** *Proliferating cell nuclear antigen* (*Pcna*), **B**. *Gene encoding protein p53,* **C.** *p53 upregulated modulator of apoptosis* (*Puma*), **D.** *Pro-caspase 3*, **E.** *Cyclin D1*, **F.** *B-cell lymphoma 2 (Bcl2)*, and **G.** *Myeloid cell leukemia 1* (*Mcl1*). Values are presented as mean ±SEM. *p < 0.05, ***p < 0.001, and ****p < 0.0001.

Subsequently, we evaluated the expression of genes related to cell proliferation and survival. Proliferating cell nuclear antigen (PCNA) is a critical DNA repair protein during DNA replication and its overexpression is correlated with colorectal carcinoma progression and metastasis^43^. We observed increased colonic expression of *Pcna* specifically in Phgg-fed mice received DSS (Fig. 4A), suggesting increased cell proliferation. Relative to control, the tumor-suppressing protein *p53* and its effector protein, p53 upregulated modulator of apoptosis (*Puma*), exhibited significantly decreased expression with Phgg consumption (Fig. 4B-C). However, their mRNA transcripts remained unchanged in inflammatory conditions induced by DSS. Alongside, the colonic mRNA level of *caspase3*, a potent inducer of apoptosis, was reduced in the Phgg-DSS group compared to the Con-DSS group (Fig. 4D). The mRNA levels of cell survival-related genes such as *cyclin D1*, B-cell lymphoma 2 (*Bcl2*), and myeloid leukemia 1 (*Mcl1*) remained unaltered across all groups (Fig. 4E-G). Collectively, these results indicate that Phgg distinctively alters the colonic expression of genes favoring barrier dysfunction and cell proliferation, particularly in the inflamed environment.

### 2.4. Phgg promotes colitis-associated colon tumorigenesis in both male and female mice

**Figure 5:**
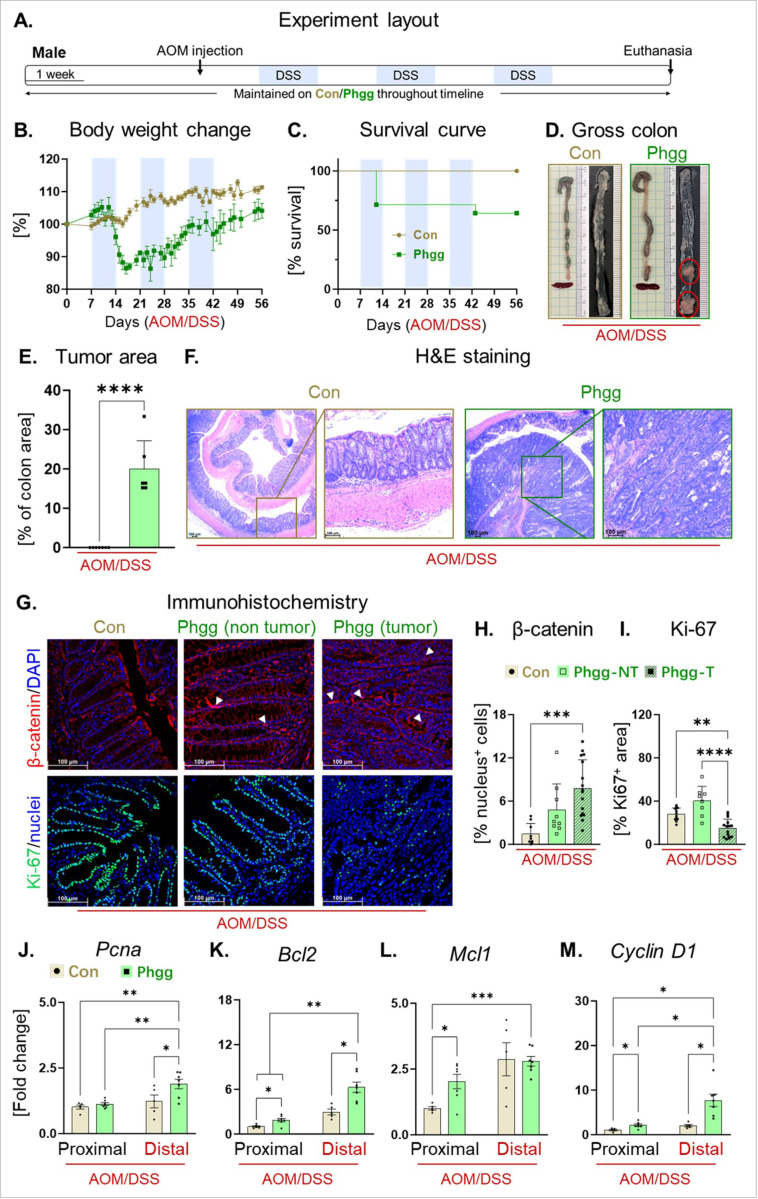
Phgg exacerbated colitis-associated colorectal cancer in AOM/DSS male mice. **A.** Experimental timeline, mice were maintained on either Phgg-contained or the control diet. Azoxymethane was administrated (AOM, 7.5 mg/kg body weight, i.p.) 1 week before three 7-day cycles of DSS/regular water (1%, 0.75%, 0.75% of DSS in drinking water), then sacrificed after one more week of regular water. **B.** Changes in body weight referred to the weight on AOM injection day. **C.** Probability of survival. **D.** Representative pictures of gross colons. **E.** Tumor area as percentage of colon area. **F.** Representative H&E-stained colon sections (original magnification, x100). **G.** Representative images of β-catenin (red) and Ki67 (green) immunohistochemical staining. DAPI was used to visualize nucleus [blue, (original magnification, x200)]. Quantitative evaluation of **H.** β-catenin (nuclear localization) and **I**. Ki-67 positive cells normalized per unit area. (**J-M**) Colonic mRNA levels of **J.** *Pcna,* **K.** *Bcl2*, **L.** *Mcl1*, and **M.** *Cyclin D1*. Values are presented as mean ±SEM. *p< 0.05, **p < 0.01, ***p < 0.001, and ****p < 0.0001.

As demonstrated in the previous section, the Phgg-fed group displayed a distinctive increase in the chemokines and cytokines linked with colon carcinogenesis. Additionally, we observed elevated expression of cell proliferation marker and reduced levels of tumor suppressor and anti-apoptotic genes. Therefore, we hypothesized that Phgg supplementation may potentiate CAC development. To test this, WT male mice were fed either a control or Phgg-containing diet for four weeks and then received a single injection of AOM (7.5 mg/kg body weight). After one-week, colonic inflammation was instigated with 1% DSS, followed by two additional cycles of 0.75% w/v DSS (Fig. 5A). Since Phgg-fed mice developed extensive colitis even at a reduced dose of DSS (1.4% w/v), we used an even lower dose of DSS in the AOM/DSS model. Despite using the very low dose of DSS, the Phgg group exhibited a substantial loss of body weight particularly during DSS administration phase (Fig. 5B). Most notably, 3 out of 7 (~ 43% of total number) Phgg-fed mice succumbed to death due to severe colitis disease (Fig. 5C). All surviving mice on Phgg diets developed colon tumors, while no visible tumor was found in the control groups (Fig. 5D-E). To further investigate the significant finding that only Phgg-fed mice developed colon tumors, we conducted histochemical staining in colon sections. Histological examination revealed that colon tumors in the distal colon regions of Phgg-fed groups exhibit characteristics of adenocarcinoma (Fig. 5F)^44^. Additionally, we observed a significant increase in nuclear translocation of β-catenin in the tumor regions compared to the control group (Fig. 5G). This increased nuclear β-catenin suggests activation of the Wnt-β-catenin signaling pathway^45^ upon Phgg feeding. Interestingly, the adjacent non-tumor regions of the Phgg-fed groups also exhibited a moderate increase in β-catenin nuclear localization compared to the control group, suggesting a potential predisposition to tumorigenesis. Surprisingly, expression of Ki-67, a cell proliferation marker^46^, was reduced in the tumor regions compared to adjacent normal tissue in the Phgg group and the control group (Fig. 5G-I). This finding aligns with previous studies demonstrating that reduced Ki-67 expression is associated with later stages of CRC^47^ and lower survival rates^48^.

To mechanistically understand how Phgg supplementation induced extensive colon tumorigenesis, we subsequently examined the expression levels of genes regulating cell proliferation and apoptosis in both the control and Phgg-fed groups. Since the proximal colon region in the AOM/DSS model did not exhibit tumorigenesis, we selected this region to represent the non-tumor bearing area. The distal colon region displayed extensive tumorigenesis, especially in the Phgg-fed group, therefore, we selected distal colon to represent tumor-prone area. Remarkably, the distal colon displayed increased mRNA transcripts of *Pcna* exclusively in Phgg-fed group (Fig. 5J). Furthermore, colonic expression of *Bcl2*, whose overexpression is known to suppress apoptosis and promote cell survival^49^, was significantly elevated in the Phgg-fed group that received AOM/DSS (Fig. 5K). The expression of *Mcl1*, a pro-survival member of the *Bcl2* protein family^50^, was also augmented in the proximal region of Phgg-fed group (Fig. 5L). Moreover, both proximal and distal region of Phgg-fed group displayed increased expression of *cyclin D1*, a regulator of cell cycle progression (Fig. 5M). The increased expression of genes suppressing apoptosis, promoting survival, and regulating cell cycle progression in both proximal and distal regions (Fig. 5K-M) suggest that Phgg supplementation may contribute to a pro-tumorigenic environment, leading to extensive tumorigenesis in the AOM/DSS treated mice.

**Figure 6:**
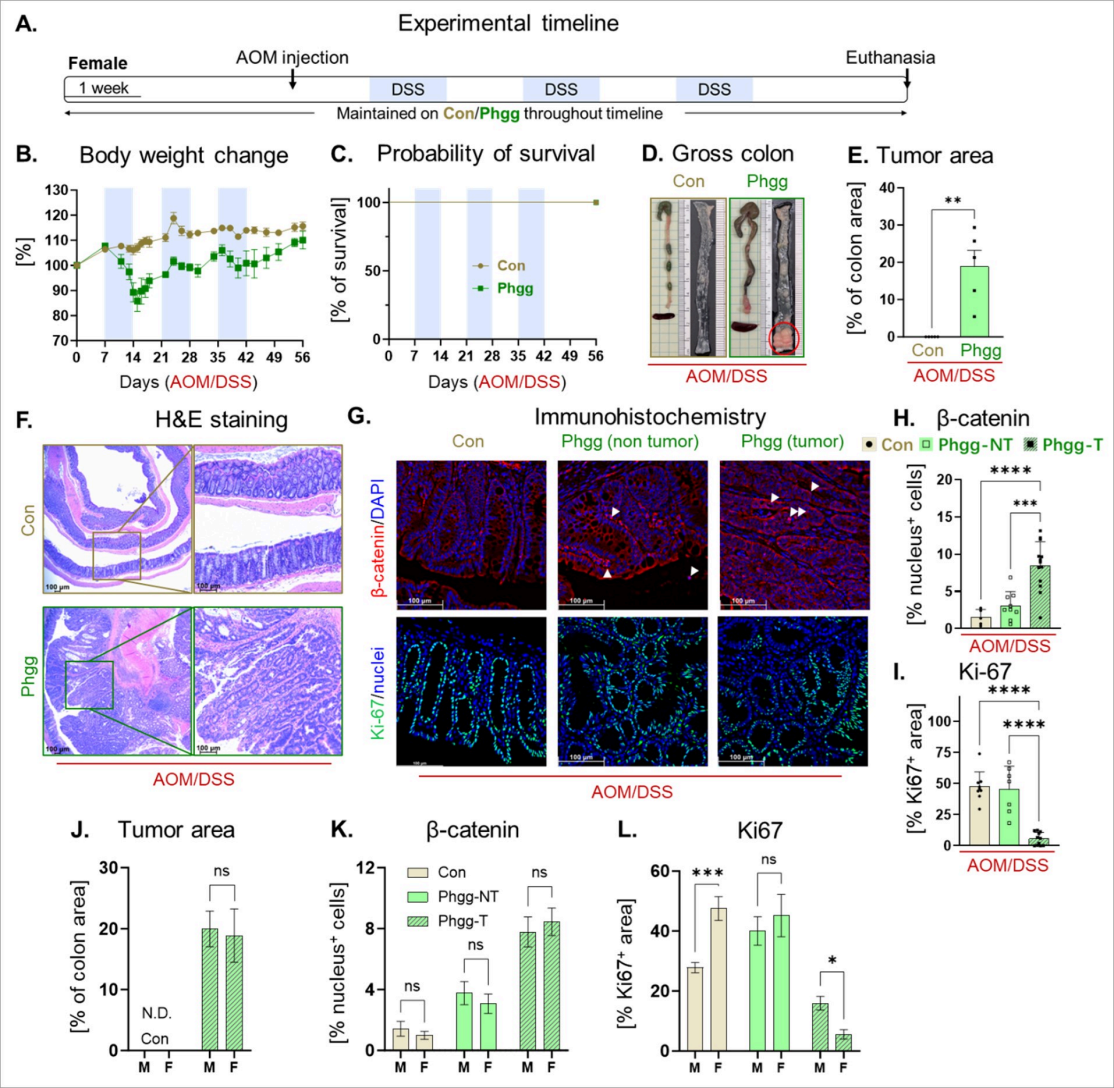
Phgg-fed female mice exhibited colon tumorigenesis comparable to that of male mice **A.** Experimental timeline. **B.** Change of body weight referred to the weight on AOM injection day. **C.** Probability of survival. **D.** Representative images of gross colon. **E.** Tumor area in percentage of the total colon surface area. **F**. Representative images on H&E staining (original magnification, x100). **G.** Representative images of β-catenin (red) and Ki67 (green) immunohistochemical staining counterstained with DAPI (blue; original magnification, x200). Quantitative evaluation of **H.** β-catenin (nuclear localization) and **I.** Ki-67 positive cells normalized per unit area. **(J-L)**Comparative analysis of colon tumor area, β-catenin-positive nuclei, and Ki67 levels between male and female cohort. **J.** % tumor area. **K.** β-catenin (nuclear localization). **L.** Ki-67 positive cells. Values are presented as mean ±SEM. *p< 0.05, **p < 0.01, ***p < 0.001, and ****p < 0.0001.

Gender-based differences in CRC incidence have been observed in humans, with males showing a relatively higher occurrence than females^51^. To assess whether Phgg feeding display similar pattern in CAC development, we next investigated colon tumorigenesis in female cohort under a similar intervention. Similar to male mice, Phgg-fed female mice experienced more body weight loss and developed extensive colon tumors after AOM/DSS treatment (Fig. 6A-F). Remarkably, control diet-fed female mice treated with AOM/DSS did not develop any visible colon tumors. As evidenced by histochemical staining, colon tumors in Phgg-fed female mice invaded the submucosa and exhibited increased nuclear localization of β-catenin and decreased levels of Ki67 expression in the tumor region (Fig. 6F-I). Our comparative analysis of colon tumor area, β-catenin-positive nuclei, and Ki67 levels revealed comparable tumorigenesis in both male and female mice (Fig. 6J-L). Taken together, these results suggest that PHGG supplementation increased susceptibility to colitis and promoted colon tumorigenesis in both male and female mice.

### 2.5. Diet with lower amount of Phgg also exacerbated colonic inflammation and carcinogenesis.

**Figure 7:**
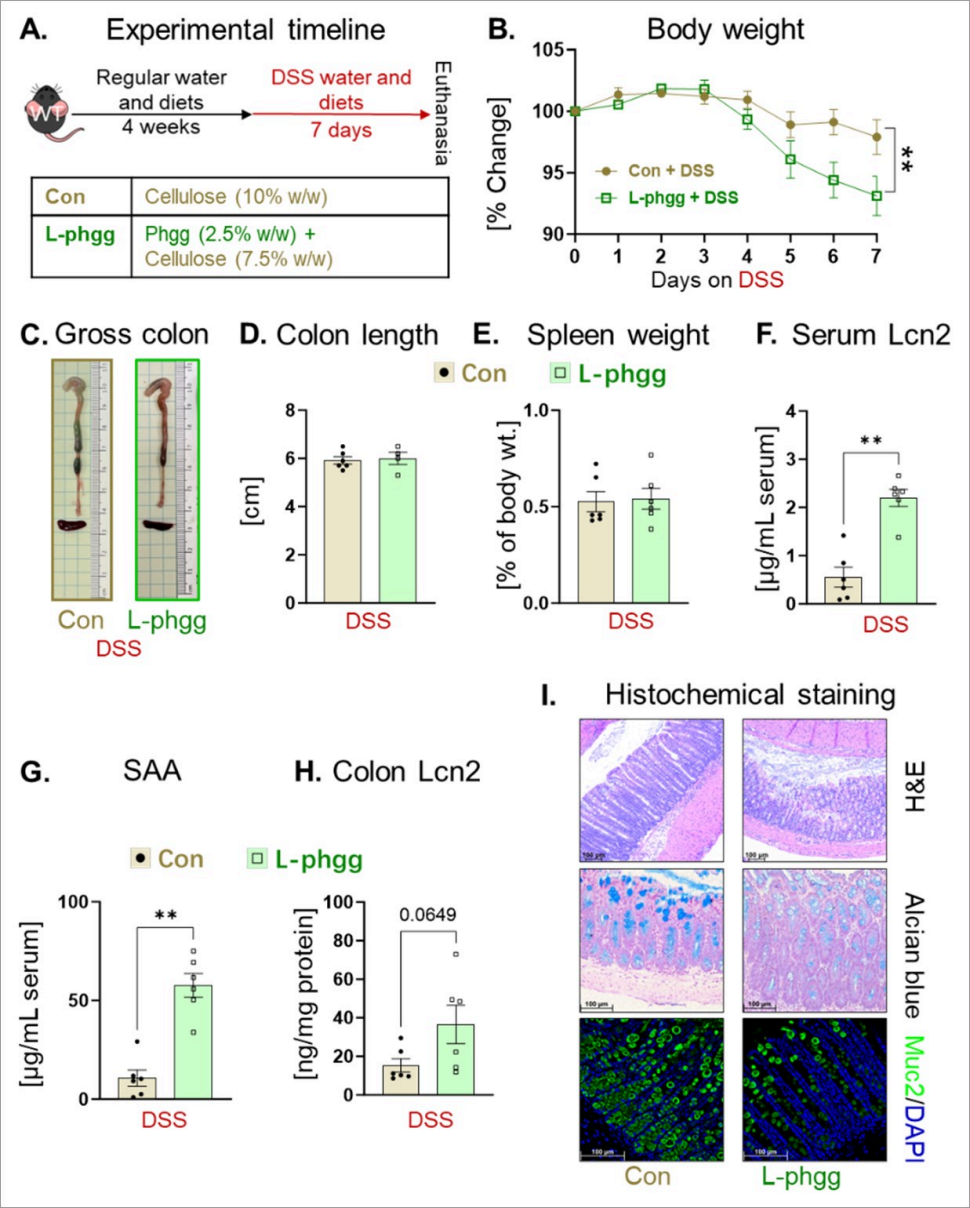
Diet with lower Phgg (L-Phgg) also exhibited augmented colitis. **A.** Experimental timeline: Four-week-old WT mice were maintained on either a control (Con) or Low-Phgg diet for 4 weeks. Afterwards, both groups received 1.4% (w/v) DSS in their drinking water for 7 days. **B.** Percent change in body weight. **C.** Gross colon appearance. **D.** Colon length. **E.** Spleen weight as percent of body weight. Serum levels of **F.** SAA and **G.** Lcn2. **H.** Colonic level Lcn2. **I.** Representative images of H&E, Alcian blue (original magnification, x100), and Muc2 (green, original magnification, x200) staining. Values are presented as mean ±SEM. *p< 0.05, **p < 0.01, ***p < 0.001, and ****p < 0.0001.

To investigate whether a reduced intake of Phgg has distinct effects on colitis and CAC development, we formulated a low-Phgg diet (L-Phgg) containing one-third amount of Phgg (2.5% w/w) than regular Phgg diet used in this study. To maintain equivalent total fiber content (10% w/w), we proportionally increased the cellulose content from 2.5–7.5% in the low-phgg diet. Similar to Phgg study, four-week-old WT mice were placed on either control (Con) or low Phgg diet (L-phgg; 2.5% w/w Phgg and 7.5% w/w cellulose) diet for four weeks. Then both groups were transitioned to a DSS (1.4%) containing water for 7 days (Fig. 7A). Low Phgg consumption exacerbated colonic inflammation, as evidenced by a 4% greater loss in body weight compared to the control group, elevated serum Lcn2 and SAA (Fig. 7B-G). The L-Phgg group showed an increasing trend in colonic Lcn2 levels, but did not reach statistical significance (p = 0.0649) (Fig. 7H). Histological analysis further supported the increased severity of colonic inflammation, showing more disrupted crypt structure and reduced level of mucin in L-Phgg group than control (Fig. 7I).

**Figure 8:**
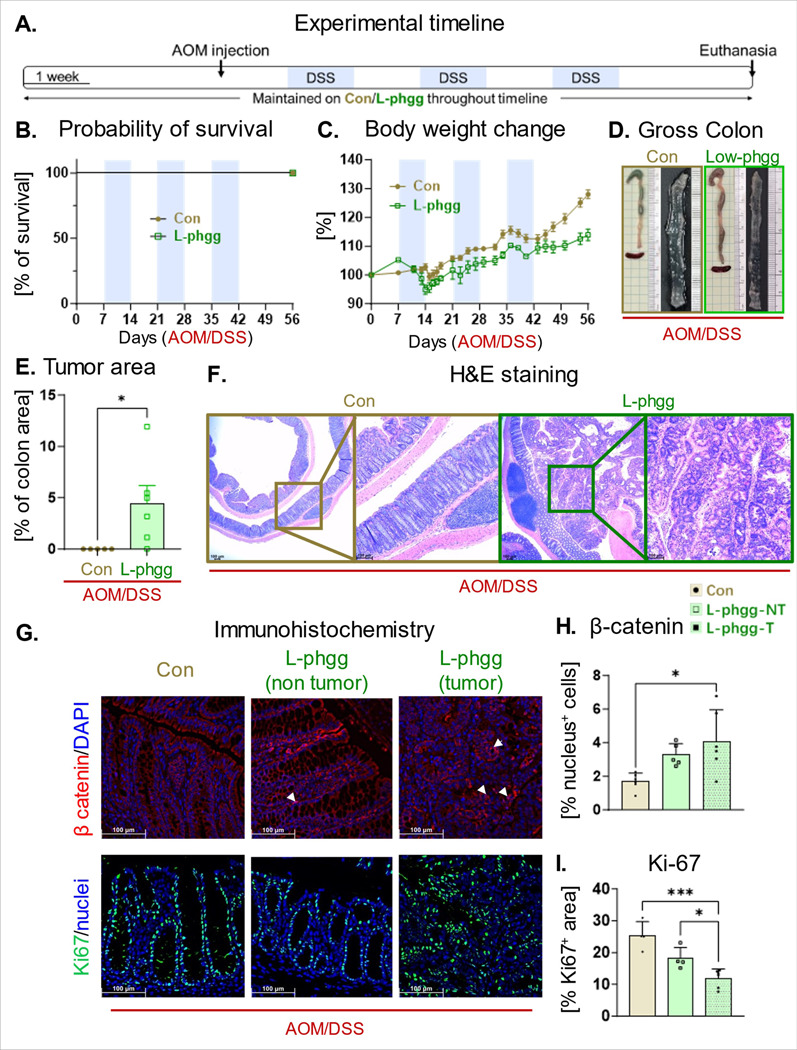
Diet containing low amount of Phgg also promoted colon tumorigenesis. **A.** Experimental timeline. **B.** Probability of survival. **C.** Percent change in body weight. **D.** Representative gross colon images. **E.** Tumor occupied area as a percentage of total colon area. **F.** Representative images of H&E-stained colon sections (original magnification, x100). **G.** Representative images displaying immunohistochemical staining for β-catenin (red, upper panel) and Ki67 (green) DAPI was used to visualize nucleus [blue, (original magnification, x200)]. Quantitative evaluation of **H.** β-catenin (nuclear localization) and **I.** Ki-67 positive cells normalized per unit area Values are presented as mean ±SEM. *p< 0.05, and ***p < 0.001.

To elucidate how this lower dose of Phgg impacts colon tumorigenesis, we employed the AOM/DSS model as described previously (Fig. 8A). The L-Phgg group consistently exhibited lower body wt, however, no mortality was observed in this group (Fig. 8B-C). Notably, the L-Phgg group also exhibited colon tumorigenesis, although tumors were not visually apparent. Histological analysis confirmed the presence of colorectal polyps (Fig. 8E-F). Indeed, the tumor-occupied colon area in the L-Phgg group was substantially lower (~ 5% of total colon area) than the Phgg-fed group (~ 20% of total colon area). Immunohistochemical analysis revealed an increase in β-catenin nuclear localization and a decrease in Ki67 expression in the tumor regions, mirroring the trends observed in the Phgg-fed AOM/DSS cohort (Fig. 8G-I). Most notably, no visible tumor was observed in the control group received AOM/DSS (Fig. 8).

## 3. Discussion

Emerging studies indicate that the effect of DFs on intestinal health varies depending on the presence or absence of ongoing inflammation. In particular, soluble DFs generally have beneficial effects on gastrointestinal health^52, 53, 54, 55, 56^ in individuals with healthy gut. However, these DFs may have adverse effects on clinical outcomes in patients with IBD ^20, 57, 58^. Our study demonstrates that Phgg has no adverse effects in healthy mice without colonic inflammation. However, in the experimental group with ongoing inflammation, Phgg exacerbated colonic inflammation and induced extensive colon tumorigenesis. Mechanistically, Phgg supplementation induced imbalanced alterations in intestinal immune activity, favoring inflammation by increasing the production of pro-inflammatory chemokines and cytokines and by suppressing the release of anti-inflammatory cytokines. Additionally, mice fed a Phgg-supplemented diet exhibited aberrant expression of genes regulating gut barrier function, cell proliferation, apoptosis, and tumor suppression, leading to increased susceptibility to CAC in the AOM/DSS model.

The Dietary Guidelines for Americans 2020–2025 recommend 28–34 grams of total fiber intake for men and women, while the average dietary fiber intake of Americans is ~ 15 grams per day^7^. DF supplements offer a convenient way to meet the recommended daily intake. As a result, these supplements have become a popular choice for bridging nutritional gaps^59^. Unlike natural sources of DF like fruits and vegetables, which contain a variety of fibers such as cellulose, hemicellulose, pectin, and lignin, DF supplements often consist of a single fiber type. Our understanding of the effects of mono-fiber supplementation on gastrointestinal health, particularly in inflamed intestines, remains limited. Experimental findings from our mouse model study indicate that Phgg, a prebiotic supplement, may not benefit intestinal health, as it increased susceptibility to colonic inflammation and promoted colon tumorigenesis. To understand the underlying mechanisms, we examined the colonic expression of inflammatory proteins which play a critical role in the progression and development of intestinal inflammation and CAC. CXCL1, a member of the C-X-C chemokine family, induces chemotaxis and infiltration of immune cells, primarily neutrophils, and contributes to the development of colitis^60^. Cxcl1 signals through G protein-coupled chemokine receptor Cxcr2 and is shown to promote tumor growth, proliferation, and metastasis of malignant cells in CRC^61, 62^. The Phgg-fed group exhibited elevated colonic levels of Cxcl1, both at the mRNA and protein levels, suggesting its potential role in the Phgg-induced exacerbation of colitis and CAC development. IL-6 and IL-1β, key regulators of chronic intestinal inflammation, play roles in both colitis and colon tumorigenesis^63, 64, 65, 66^. IL-6 activates STAT3 to promote tumor initiation and growth^67^ and drives Fos-related antigen 1(FOSL1/ FRA1) deacetylation, endowing CRC cells with stem cell-like properties and enhancing their proliferation^68^. The inflammatory cytokine IL-1β has also been shown to mediate colonic inflammation^65^ and facilitate the stemness and invasiveness of CRC cells through the epithelial-mesenchymal transition activator zinc finger E-box binding homeobox 1 ^66^. In our study, elevated levels of these inflammatory cytokines were observed along with Cxcl1 in mice fed Phgg, suggesting a collective role of chemokines and cytokines in the Phgg-mediated exacerbation of intestinal inflammation and colon carcinogenesis. Inflammatory cytokines including IL-6 and IL-1β increase intestinal permeability^69, 70, 71^. In agreement, we observed aberrant expression of tight junction (TJ) proteins in Phgg-fed groups received DSS. Similar to what observed in human colon carcinoma tissues^72, 73^, we found increased colonic mRNA levels of claudin-1 and − 2, and decreased claudin-7, in the Phgg-fed group. Increased expression of claudin-1 and − 2, coupled with reduced claudin-7 has been observed in IBD^39^. Additionally, elevated colonic levels of both claudin-1 and - 2 are known to be linked to increased colon tumorigenesis in humans^74, 75, 76, 77^.

PCNA is a crucial protein for the development of colorectal cancer by participating in DNA replication and repair, which are essential for tumor growth and progression. PCNA acts as a sliding clamp for DNA polymerase during the synthesis of new DNA strands, ensuring accurate replication during the S-phase of the cell cycle. In CRC, PCNA is often overexpressed, and this heightened expression correlates with increased cellular proliferation and more aggressive cancer characteristics^78^. Additionally, PCNA is closely associated with Cyclin D1, a regulator of the G1-to-S phase transition in the cell cycle, as both proteins are involved in regulating the cell cycle and ensuring efficient DNA replication during tumor growth^79, 80^. Notably, the Phgg + DSS groups showed elevated mRNA expression of both *Pcna* and *Cyclin D1*, indicating that Phgg-induced changes in cell proliferation regulators potentiating CRC development in the AOM/DSS group. Moreover, *Mcl1* (myeloid cell leukemia-1) is a widely recognized pro-survival member of the *Bcl2* (B-cell lymphoma protein 2) and notably recognized for anti-apoptotic role in the Bcl-2 family^50^. The colonic expression of both *Mcl1* and *Bcl2* was increased in the Phgg-fed group received AOM/DSS. Collectively, the increased expression of genes regulating proliferation and pro-survival specifically in the proximal region indicates that Phgg supplementation promoting a pro-tumorigenic environment, leading to extensive tumorigenesis in the AOM/DSS treated mice. The colonic tumors observed in Phgg-fed group displayed a significant increase in nuclear translocation of β-catenin, indicating an activation of the Wnt-β-catenin signaling pathway. Interestingly, a moderate increase in β-catenin nuclear localization was also observed in the adjacent non-tumor regions of the Phgg-fed groups compared to the control, implying a possible predisposition to tumorigenesis. The observations from human CRC cohorts show high expression of Ki67, a cell proliferation marker, in colon tumor region linked to improved clinical outcome^48, 81, 82^. We observed reduced expression of Ki-67 in the tumor regions compared to adjacent normal tissues. A longer-term study will help investigate whether reduced Ki67 expression in the Phgg-fed group is linked to increased colon tumor burden and the development of advanced colorectal neoplasms.

In summary, our findings suggest potential risks associated with isolated Phgg consumption, particularly as a supplement for individuals with ongoing intestinal inflammation. Further research is necessary to elucidate the intricate interplay between mono-DF supplements and host intestinal health, especially in the context of IBD. Personalized nutrition strategies that account for individual health conditions are crucial for optimizing the types and amounts of DF consumed to minimize adverse outcomes.

## 4. Methods

### Mice and diets

C57BL/6 wild-type (WT) mice were bred and maintained under specific pathogen-free conditions in a humidity- and temperature-controlled room at The Pennsylvania State University in University Park, Pennsylvania. At four weeks of age mice were divided into experimental groups. Throughout the study, mice had unrestricted access to experimental diets (control, Phgg-containing diet, or low-Phgg diet) and water. The diets were prepared by Research Diets, Inc. (New Brunswick, NJ). A detailed composition of all three diets is provided in table 1. Food, water, and cages were replaced weekly. All procedures complied with the guidelines of the Institutional Animal Care and Use Committee of Pennsylvania State University

### DSS-induced colitis study

Four-week-old C57BL/6 wild-type mice were fed control or Phgg-containing diets for four weeks. These mice were then randomly divided into two groups: a basal-feeding group (NT) and a colitis group. Colitis was induced by administering drinking water containing 1.4% w/v dextran sulfate sodium (DSS, MP Biomedicals) for seven days. Daily body weights were monitored throughout the DSS period. The NT groups receiving control or Phgg-diet were maintained on water only.

### Colitis-associated colon cancer study

Four-week-old WT mice were fed with either control or Phgg-diet. After four weeks of diet feeding both control and Phgg-diet received a single dose of azoxymethane (AOM, 7.5 mg/kg, i.p.). One week later, DSS/regular water cycles began, starting with 1.0% DSS water and then decreasing to 0.75% for the remaining two cycles. Each cycle consisted of 14 days, with 7 days of DSS water followed by 7 days of regular water. The mice were euthanized one week after completing the final cycle. All mice continued their assigned diets throughout the experiment and were monitored for their body weights regularly.

### Sample collection and preparation

Upon completion, mice were humanely euthanized using CO_2_. Blood was collected into serum-separation tubes, followed by centrifugation at 8000 g for 8 minutes at room temperature. The serum was then stored at −80°C until analysis. Colon segments were snap-frozen in liquid nitrogen, collected in RNAlater solution, or fixed in 10% neutral buffered formalin (NBF) for cytokine/chemokine measurement via ELISA, mRNA expression via qPCR, and histochemical staining, respectively.

### RNA isolation and quantitative polymerase chain reaction (qPCR)

The colon tissues were homogenized, and total RNA was extracted using TRIZOL reagent (Invitrogen) following the manufacturer’s instructions. The concentration of the extracted RNA was estimated with a NanoDrop spectrophotometer (Thermo Scientific). The RNA obtained from DSS-treated groups were purified *via* lithium chloride precipitation method. RNA was then converted to complementary DNA with ScriptTM XLT cDNA supermix kit (QuantaBio) according to the manufacturer’s instructions. qPCR was performed to assess the expression of colonic genes encoding inflammatory, proliferative, apoptotic, and tight junction markers using SYBR Green master mix (Thermo Fisher) on QuantStudio 3 Real-Time PCR System (Applied Biosystems). Relative expression levels were calculated by 2^−ΔΔCt^, with 36B4 as an endogenous reference for normalization. A list of the primers is provided in Table 2.

### Histochemical analysis

For histochemical staining, colon tissue was dissected from the mice and feces was flushed with ice cold PBS to clean the colon and Swiss roll was prepared and fixed in 10% neutral buffered formalin (NBF) for 24 hours. After that, the Swiss roll tissue was transferred to 70% ethanol. Further, the colon was dehydrated using an alcohol gradient (70%, 90%, 95%, and 100%), followed by two treatments with xylene. The tissue was then embedded in paraffin wax and sectioned (thickness 5μM) using a microtome. The paraffin-embedded tissue sections were deparaffinized in xylene and rehydrated with a gradient of alcohol (100%, 95%, 70%, and 50%) using a Leica Autostainer XL (Leica Biosystems).

#### Hematoxylin and Eosin (H&E) staining

H&E staining was performed by the animal diagnostic laboratory, The Pennsylvania State University, and imaged by a Leica DMi8 microscope, Leica Microsystems.

#### Alcian blue staining

The colon sections were deparaffinized using a Leica Autostainer XL (Leica Biosystems) and then stained with Alcian blue (Vector Laboratories Inc) following the manufacturer’s instructions to evaluate goblet cell-containing acidic mucus.

### Immunofluorescence staining

Immunohistochemical staining was performed as described in our previous studies ^21, 23^. Briefly, deparaffinized sections were incubated in pre-warmed sodium citrate buffer (pH 6.0) at 98°C for 20 minutes in a water bath, followed by washing with PBS. To block the non-specific sites, the tissue sections were then incubated with 10% donkey serum containing 0.3% Triton X-100 (VWR Life Sciences) for 90 minutes at room temperature. Subsequently, the sections were incubated overnight at 4°C with primary antibodies [β-catenin (Novus Biologicals); Ki67 (Novus Biologicals); Muc2 (Abcam)] diluted in PBS containing 1% donkey serum (Sigma-Aldrich), 1% bovine serum albumin (BSA, Sigma-Aldrich), and 0.3% Triton X-100. After incubation, the sections were washed with PBS three times and then incubated with the secondary antibody [anti-mouse Alexa Fluor^™^ 555 (β-catenin) and anti-rabbit Alexa Fluor^™^ 488 (Ki67)] for 90 minutes at room temperature in a dark chamber, followed by mounting with antifade reagent containing 4’,6’-diamidino-2-phenylindole and an anti-fading agent (Sigma Fluoroshield^™^, F6057) for nucleus staining. All histological images were captured using the Leica DMi8 with the LAS X software (Leica Microsystems Inc.) and quantified using ImageJ software.

### Image Quantification

The colon tumor area, Ki67 expression, and β-catenin localization were quantified using ImageJ software with the Fiji extension. The entire colon area and the tumor area were automatically identified through color-based selection. Ki-67 expression was estimated by the stained area in the green channel (Ki-67) normalized by the stained area in the blue channel (nucleus). The nuclear localization of β-catenin was identified by counting the number of overlapping areas between the red channel (β-catenin) and the blue channel (nucleus) after filtering with watershed. The number of nuclei was counted by the number of areas with a circularity greater than 90% for an ellipse after filtering with watershed in the blue channel. Both numbers were counted using the particle measure function. The number of β-catenin-positive nuclei was then normalized by the total number of nuclei.

### Statistical Analysis

All data are presented as Mean ± S.E.M. The assessment of normality and equal variance was conducted using the Shapiro–Wilk and Bartlett tests within RStudio. Statistical significance between the two groups was determined via an unpaired, two-tailed t-test (parametric) or an unpaired non-parametric Mann–Whitney test. *p* value style: 0.05 (*), 0.01 (**), < 0.01 (***). Comparisons involving more than two groups, one-way ANOVA or Welch and Brown-Forsyth ANOVA were applied for parametric and non-parametric data, respectively, followed by Tukey’s multiple comparison tests. *p* value style: 0.05 (*), 0.01(**), 0.001 (***), < 0.0001 (****).

## Supplementary Material

Supplement 1

## Data Availability

The original data generated in this study is included in this article. Further inquiries can be directed to the corresponding author.
