# Supplementary material for: Processed Dietary Fiber Partially Hydrolyzed Guar Gum Increases Susceptibility to Colitis and Colon Tumorigenesis in Mice
Source: Res Sq. 2024 Dec 9:rs.3.rs-5522559. Preprint. [Version 1] doi: 10.21203/rs.3.rs-5522559/v1 (PMC11661293; doi:10.21203/rs.3.rs-5522559/v1)
Supplement: Supplement 1 [file NIHPPRS5522559V1-supplement-1.pdf]

Supplementary Files

This is a list of supplementary files associated with this preprint. Click to download.

- Table12.docx
